# Sudden Death Due to Giant Cell Myocarditis: A Case Report

**DOI:** 10.14740/cr446e

**Published:** 2015-12-16

**Authors:** Janani Shanmugam, Paranthaman Sampath Kumar, Vinod Kumar Panicker, Prathiba Duvooru

**Affiliations:** a Department of Forensic Medicine & Toxicology, Sri Ramachandra Medical College & Research Institute, Sri Ramachandra University, Chennai, Tamil Nadu, India; b Department of Transfusion Medicine, Sri Ramachandra Medical College & Research Institute, Sri Ramachandra University, Chennai, Tamil Nadu, India; c Department of Pathology, Sri Ramachandra Medical College & Research Institute, Sri Ramachandra University, Chennai, Tamil Nadu, India

**Keywords:** Sudden cardiac death, Giant cell myocarditis, Autopsy

## Abstract

The definition of sudden death is variable and there has been no single all-purpose definition. Sudden death can be defined as sudden or unexpected death in an otherwise healthy individual who is not known to have been suffering from any dangerous disease, injury or poisoning and is found dead or dies within 24 hours after the onset of terminal illness. Some authorities limit the duration to 1 hour. Because of the sudden and unexpected nature of death, inquest is conducted in such cases to rule out foul play and ascertain the cause of death. A vast majority of cases are due to cardiac origin followed by respiratory, neurological, gastrointestinal and genitourinary causes. The most common cardiac disease to cause sudden death is ischemic heart disease as a result of coronary atherosclerosis. Coronary artery disease, cardiomyopathies and electrophysiologic abnormalities are the common causes of sudden cardiac deaths. We present a rare case of sudden death in a healthy adult male due to giant cell myocarditis.

## Introduction

Numerous definitions have been proposed for sudden cardiac death (SCD) in the past and definition of the word “sudden” has been debated [1]. “An unexpected death from a cardiovascular cause in a person with or without preexisting heart disease” is usually called SCD. In literature, most of the cases are associated with a sudden collapse, death occurring within 1 h of onset of symptoms, or an unexpected death that occurred within the previous 24 h [2-5]. SCD is a major public health problem. There is increased incidence of SCD in developing countries including India. About 40-50% of all cardiovascular deaths have been attributed to SCDs and about 80% of these are caused by cardiac arrhythmias. Cardiovascular diseases (CVDs) are a major cause of global mortality, responsible for almost 17 million deaths every year. In developing countries, it is responsible for twice as many deaths as human immunodeficiency virus (HIV), malaria and tuberculosis (TB) combined. About 6 million SCDs occur every year due to cardiac arrhythmias. Cardiovascular mortality has been considerably reduced in the US due to increased awareness of risk factors and prevention of CVD. However, it still poses a huge burden in India and abroad. Hence, there is an urgent need to develop strategies for management of SCD. The incidence of SCD increases markedly with age. The most common electrical event with SCD is progression of ventricular tachycardia (VT) to ventricular fibrillation [6].

Accurate statistics regarding the current incidence of SCDs in India are unavailable and unreliable as most of the data are collected by verbal autopsy where a number of cases could be missed or misdiagnosed. Also clinical autopsies are done very rarely where the actual cause of death could be ascertained. Moreover, a number of deaths occur at home even before people reach hospitals which include deaths in rural areas where people have limited access to hospitals. In a questionnaire-based study that involved medical students from South India, SCD was responsible for about 10.3% of mortality in the population from Southern India. On an average, the SCD cases were found to be 5 - 8 years younger when compared to population from the west. Also major risk factors for coronary artery disease were highly prevalent [7]. Performing autopsies in sudden and unexpected deaths gives a specific, objective and scientific cause of death. Coronary artery disease, cardiomyopathies and electrophysiologic abnormalities are the predominant causes of SCDs [6-9]. Myocarditis as a cause of sudden death has been quoted in literature [10-12]. Idiopathic giant cell myocarditis (IGCM) is an extremely rare but well-known fatal entity presenting with arrhythmias and heart failure [13, 14]. Few cases of IGCM presenting as sudden death and diagnosed at autopsy have been reported in the past [15-19]. We present a case of sudden death in an otherwise healthy male due to IGCM.

## Case Report

A 33-year-old male, working in a departmental store, fainted at around 12 noon while talking at his workplace as per eyewitness’ history. He had travelled by bus for about 6 h the previous night and reported to work after his breakfast. He was rushed to the hospital by his co-workers where he was declared brought dead within an hour of the incidence. There was no history of previous episodes or co-morbidities. There was no family history of sudden deaths. His body was shifted to the mortuary. After routine inquest, autopsy was conducted on the next day.

### Examination

**Figure 1:**
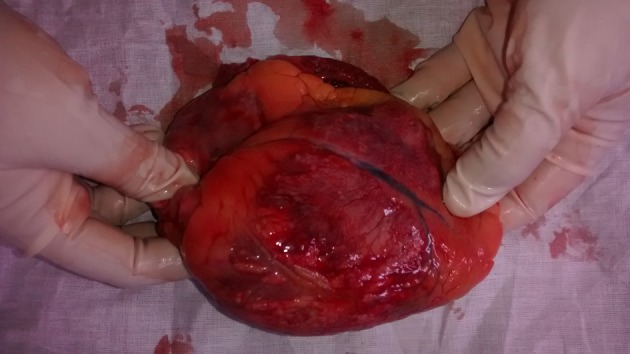
Heart showing grayish white elevated patches on the epicardium.

**Figure 2:**
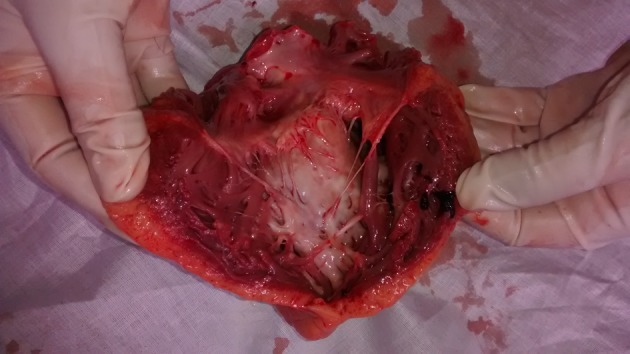
Heart showing grayish white ventricular septum.

**Figure 3:**
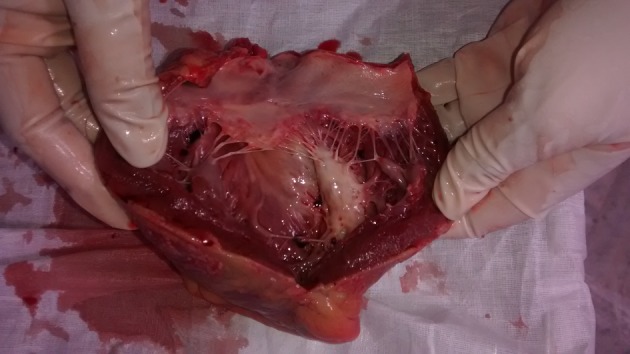
Heart showing grayish white ventricular septum and whitish papillary muscle.

**Figure 4:**
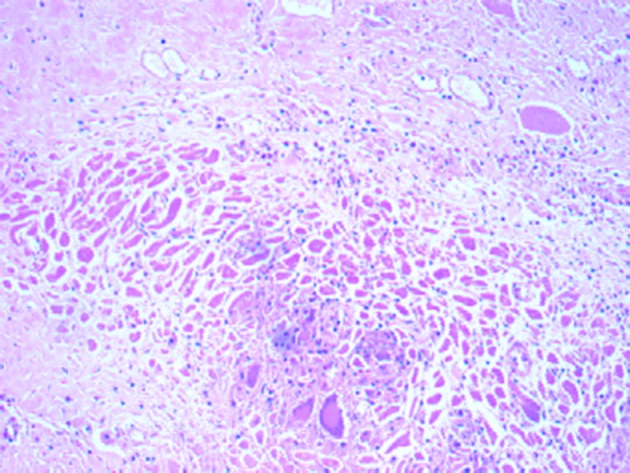
Section of myocardium showing many multinucleated giant cells (H&E, × 100).

**Figure 5:**
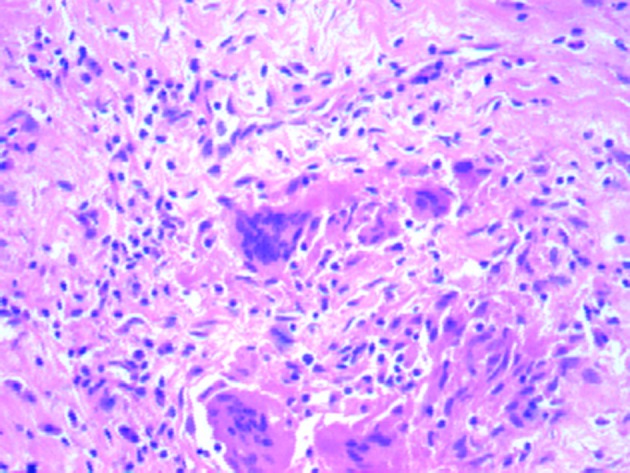
Section of myocardium showing many multinucleated giant cells (H&E, × 400).

The deceased was a well built adult male, with no external injuries on examination. On dissection, all organs were congested. Stomach contained semidigested food particles. Heart weighed 395 g. There were numerous grayish white elevated patches over the pericardial surface (Fig. 1) which was extensive on the anterior aspect. The pericardial pad of fat was congested. There were grayish white patches on the ventricular walls. Ventricular septum was grayish white (Fig. 2) as a whole and papillary muscles were whitish (Fig. 3) in color. Coronaries were patent. Histology of the heart showed areas of necrosis and fibrosis in the myocardium with chronic inflammatory cells infiltration predominantly plasma cells, few lymphocytes and eosinophils. Numerous large, multi-nucleated giant cells resembling osteoclastic giant cells were also seen. No granulomas were seen (Fig. 4, 5). Cause of death was opined as cardiogenic shock due to giant cell myocarditis.

## Discussion

CVD is the most important cause of death all over the world. About 40-50% of all cardiovascular deaths have been attributed to SCDs and about 80% of these are caused by cardiac arrhythmias. SCD is a major public health problem all over the world [6].

Inflammation of the myocardium is “myocarditis”. In previous studies, myocarditis has known to contribute to 6-12% [10, 11] of sudden cardiac deaths in young athletes. Myocarditis following an infection is common, of which viral etiology is the most common. A number of viruses including Coxsackie, hepatitis, adenovirus and HIV have known to cause myocarditis. Bacterial, fungal and protozoal infections and hypersensitivity reactions to drugs immunologic syndromes are other causes for myocarditis [20].

IGCM is a rare but known entity that presents clinically with cardiac failure or arrhythmias [13]. The incidence of IGCM is very low and variable. Of the 377,841 cases of autopsy taken from the Annuals of Autopsy Records for Japan from 1958 to 1977, 25 cases (0.007%) were recorded as IGCM [21]. It is known to be a disease of the relatively young and otherwise healthy adults [15]. The mean age in a few case series has been observed as 42.6 [14] to 57 years [13]. Few cases have also been reported in children less than the age 19 [22]. It has been identified as a distinct entity different from cardiac sarcoidosis, rheumatic heart disease and Wegener’s granulomatosis from the early 1970s [23]. The etiology is predominantly unknown. The possibility of IGCM to be caused by myocardial infection by viruses or other agents in individuals with immunological disorders has been proposed but no infective cause has been proved at autopsy yet [23]. Staining for bacilli and attempts to isolate viruses have been negative [19, 23]. In the past, IGCM has been reported in individuals with autoimmune disorders like discoid lupus erythematosus, autoimmune hepatitis, myasthenia gravis, rheumatoid arthritis, Graves disease, diabetes mellitus, lymphomas and thymomas suggesting an autoimmune etiology [23-30]. However, autoimmune disorder presenting with such rapid clinical progression is unlikely [23].

Symptomatic individuals usually present with cardiac failure, acute coronary syndromes, dysrhythmias, heart block, dyspnea, fatigue, febrile illnesses, cardiogenic shock or sudden death. Non-cardiac complaints like painless hematemsis have also been reported [13, 14, 22, 30]. IGCM is one of the causes for refractory cardiac failure. Diagnosis in the living is by endomyocardial biopsy, apical-wedge sampling or histology of the explanted heart [14].

Despite recurrence in the transplanted heart and mortality after transplantation, cardiac transplantation is currently the treatment of choice. Immunosuppressive agents including steroids, azathioprine and muromonab CD3 have known to increase survival duration in the absence of transplantation [14].

Sudden deaths in otherwise healthy individuals without any history suggestive of cardiac ailments have been reported [15-19].

Serpiginous areas of necrosis and grayish patches in the myocardium allowing an easy naked eye diagnosis on gross examination are common [15, 19, 23]. Histologically ICGM is confirmed by muscle necrosis with giant cells at the margins. Within the areas of necrosis, a florid histiocytic and eosinophilic cell infiltrate has been reported with normal adjacent myocardium [22]. Inflammatory cellular infiltration within the myocardium has been reported. Along with the infiltration, multinucleated giant cells and granulomas interspersed with lymphocytes have also been reported. The presence of eosinophils has been noted in most cases. There was typical absence of sarcoid-like granuloma [14].

The minimum information available on the condition is from the various case reports and case series. Due to rarity of the condition, there are very few studies; hence there is paucity of conclusive interpretations regarding etiology, management and prognosis. Better understanding of the etiopathophysiology will enable appropriate management of the condition. In developing countries like India where endomyocardial biopsies and apical-wedge samplings are done rarely, most of the cases are either missed or diagnosed only during autopsy.
